# 

**Published:** 1875-04

**Authors:** 


### CONTENTS:

### ORIGINAL COMMUNICATIONS.

ARTICLE I.—Valedictory Address. By Prof. Thomas S. Latimer, M. D. .......... 529

ARTICLE II.—Class Address. By James M. King D. D. S. .......... 542

ARTICLE III.—Operative Dentistry. By S. J. Cobb, D. D. S .......... 548

ARTICLE IV.—Proceedings of Virginia State Dental Association. (Continued.) .......... 553

### SELECTED ARTICLES.

ARTICLE V.—Amalgams for the Teeth. By E. A. Bogue, M. D. .......... 558
ARTICLE VI.—Treatment of Mucous Polypus of the Velum Palati .......... 562
ARTICLE VII.—Injury of the Face. By J. S. Thurman, M D. .......... 564
ARTICLE VIII.—Resection of the Upper Jaw, with Preservation of Portion of Hard Palate and Incisor Teeth .......... 565

### EDITORIAL, ETC.

Practice of Dentistry in the State of South Carolina .......... 567

### MONTHLY SUMMARY.

An Unnatural Position of the Head a Cause of Death from Anæsthetics .......... 570
Manufacture of Caoutchouc from Milk Weed .......... 571
A New Operation for Cleft Palate .......... 571
Electric Light .......... 572
Chloral Hydrate and Camphor as a Local Application in Neuralgia .......... 572
An Extraordinary Case .......... 573
Fine Wire .......... 573
Acidity of the Gastric Juice .......... 574
Lymphatic Tumors of the Face .......... 574
Actual Cautery .......... 574
Headache from Eye Strain .......... 575
Assaying by the Spectroscope .......... 575
Tenotomy in the Treatment of Fracture of Lower Jaw .......... 575
Causes of Death from Petroleum Explosion .......... 575
Hydrogen Alloys .......... 576
Lacto-phosphate of Lime for Carious Teeth .......... 576
Difference in the Composition of Teeth, etc .......... 576

